# CMID: Crossmodal Image Denoising via Pixel-Wise Deep Reinforcement Learning

**DOI:** 10.3390/s24010042

**Published:** 2023-12-20

**Authors:** Yi Guo, Yuanhang Gao, Bingliang Hu, Xueming Qian, Dong Liang

**Affiliations:** 1 Xi’an Institute of Optics and Precision Mechanics, Chinese Academy of Sciences, Xi’an 710119, China; guoyi@opt.ac.cn (Y.G.); hbl@opt.ac.cn (B.H.); 2 School of Information and Communications Engineering, Xi’an Jiaotong University, Xi’an 710049, China; qianxm@mail.xjtu.edu.cn; 3 University of Chinese Academy of Sciences, Beijing 100049, China; 4 College of Computer Science and Technology, Nanjing University of Aeronautics and Astronautics, Nanjing 211106, China; gaoyuanhang@nuaa.edu.cn

**Keywords:** deep reinforcement learning, pixel-wise image processing, crossmodal image denoising, infrared image denoising, terahertz image denoising

## Abstract

Removing noise from acquired images is a crucial step in various image processing and computer vision tasks. However, the existing methods primarily focus on removing specific noise and ignore the ability to work across modalities, resulting in limited generalization performance. Inspired by the iterative procedure of image processing used by professionals, we propose a pixel-wise crossmodal image-denoising method based on deep reinforcement learning to effectively handle noise across modalities. We proposed a similarity reward to help teach an optimal action sequence to model the step-wise nature of the human processing process explicitly. In addition, We designed an action set capable of handling multiple types of noise to construct the action space, thereby achieving successful crossmodal denoising. Extensive experiments against state-of-the-art methods on publicly available RGB, infrared, and terahertz datasets demonstrate the superiority of our method in crossmodal image denoising.

## 1. Introduction

Due to the variability of the external environment and inherent limitations of a device, noise inevitably occurs during image-sensing processes [1,2], which can seriously reduce the visual quality of the image and adversely affect downstream computer vision and multimedia tasks [3]. Computer vision image-denoising research covers multiple modal images obtained by different sensing methods, such as RGB, infrared, and terahertz images. In general, we study images in each mode in isolation and tailor the image-denoising model to learn the features from their specificity to achieve excellent results. For example, in RGB image denoising, some methods based on an image noise model have been developed to suppress noise [4,5,6]. However, most of them focus on the removal of specific noise and require manual selection in terms of the parameters. While the learning-based methods [7,8,9,10,11] achieve impressive performance by providing a series of potential heuristic constraints for training, they ignore the ability to work across modalities. Compared with RGB images, infrared and terahertz imaging has more information reduction and more serious noise interference, severely limiting its application in downstream tasks [12,13]. Although some methods attempt to denoise these images [14,15,16], training is difficult due to the lack of available datasets, and their performance is limited. We believe that the first step towards universal image denoising is to build a model that works across modalities rather than over-optimizing image denoising for one modality. Therefore, we train a crossmodal image denoising model on the relatively easy-to-obtain RGB image dataset, which can effectively achieve infrared and terahertz image denoising.

**Figure 1:**
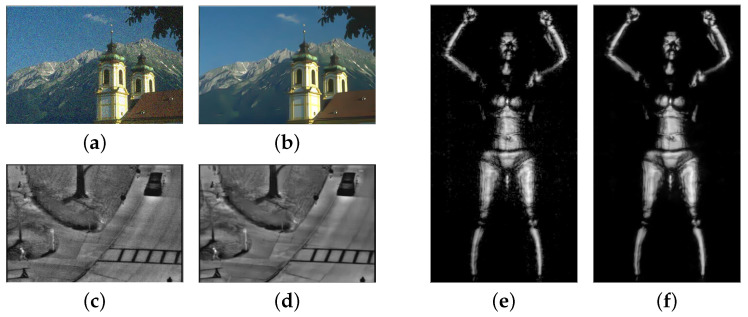
Noisy images and the corresponding results of our method in three modalities: (**a**,**c**,**e**) represent RGB, infrared, and terahertz noise images, respectively; (**b**,**d**,**f**) represent the corresponding results.

The image denoising problem can be represented by the model: x=y−n, where *x* represents the clean image, *y* represents the noisy image, and *n* represents the noise in the image. Since it is challenging to model noise directly from the acquired noisy image, the common way is to change the model into y=x+n, that is, adding noise to a clean image to simulate the noisy image, as shown in Figure 1a. From a Bayesian perspective, although prior image modeling does play a central role in image denoising when the likelihood is known, the noise in infrared and terahertz images is difficult to model accurately. The noise in infrared images is mainly a mixed Poisson–Gaussian distribution with a complex distribution function, and terahertz images contain interference information, such as complex stripe noise and block effects [17] (as shown in Figure 1c,e). We ultimately chose to add additive white Gaussian noise (AWGN) to the clean RGB images to simulate the noisy images for training. The reason for using AWGN is two-fold: first, AWGN is a natural choice when there is no specific prior information about the source of the noise. Second, most noise in the real world can be approximated as local AWGN [8].

For human professionals, the procedure of image retouching is a series of iterative decisions. Inspired by this process, we propose a pixel-wise crossmodal image-denoising method based on deep reinforcement learning. Unlike other deep learning denoising methods, our method is interpretable because we progressively denoise by iteratively selecting a series of decisions. Specifically, we regard the task process as a Markov decision process (i.e., the agent iteratively decides the action with which to interact with the environment), treating each pixel as an agent and, finally, learning an optimal strategy to maximize the similarity reward of all pixels to obtain the best result. Figure 1 shows the denoising results of our method in three modalities. When faced with different states, the network generates different strategies to select the most appropriate action and then calculates the reward that affects the generation of the next strategy based on the reward function we set, achieving progressive crossmodal image denoising. In addition, to ensure that the complex noise in different modality images can be effectively processed, an action set that can handle multiple types of noise is designed to construct the action space. Moreover, the denoising operation can be performed repeatedly or not, ensuring that a random denoising strategy with higher flexibility is learned.

Our contributions are summarized as follows:

- We proposed a pixel-wise crossmodal image-denoising method based on deep reinforcement learning, treating the task as a Markov decision process. The denoising task is divided into policy generation and reward evaluation, which are iteratively alternated to obtain the optimal policy that maximizes the similarity reward.
- We designed an action set that can handle multiple types of noise to construct the action space, which improves the processing capabilities of images in different modalities, enhances the generalization performance, and achieves crossmodal image denoising.
- Comprehensive experiments on existing SOTA methods in terms of visual quality comparison and image quality assessment demonstrate that our model not only performs well in RGB image denoising but also significantly outperforms other methods in handling noise in infrared and terahertz images.

The remainder of this paper is organized as follows: We discuss the related work in Section 2. The details relevant to our proposed model are described in Section 3. The experimental results are presented and discussed in Section 4, and the conclusions and future work are presented in Section 5.

## 2. Related Work

### 2.1. Deep Reinforcement Learning

Following the successful attainment of human-level performance on Atari games by deep Q-network, there has been a surge of interest in deep reinforcement learning (DRL). Here, we mainly review work related to applying reinforcement learning to computer vision-related tasks, especially image processing. Cao et al. [18] introduced a face image super-resolution enhancement network, which converts a local region of the agent selected into high resolution, and then the agent selects the next region that should be enhanced. This process is repeated until the maximum time step. Yu et al. [19] proposed RL-Restore to let an agent learn a strategy in terms of selecting appropriate tools from a predefined toolbox to gradually restore damaged image quality. Park et al. [20] presented a DRL framework for color enhancement. This method combined deep reinforcement learning to propose a training scheme that relies solely on high-quality images. The proposed agent iteratively selects image processing actions, such as increasing the color saturation, and applies them to enhance the input image.

In contrast to the previous methods for processing a global image, some works try to propose pixel-wise reinforcement learning for image processing. Furuta et al. proposed PixelRL [21], a method that extended deep reinforcement learning to achieve pixel-by-pixel image restoration. By applying pixel-wise reinforcement learning, PixelRL aimed to restore and enhance images at a fine-grained level. Inspired by this, Zhang et al. [22] proposed a pixel-level low-light image enhancement method to improve the quality and visibility of low-light images by applying reinforcement learning technology at the pixel level. ALL-E [23] implemented aesthetics-guided low-light image enhancement based on pixel-wise reinforcement learning. We implement pixel-wise image denoising based on the reinforcement learning framework and design an action set that can handle a variety of noises to achieve crossmodal image denoising.

### 2.2. Image Denoising

Since training samples of RGB image denoising are plentiful and easy to obtain, RGB image denoising has already been extensively studied for several decades. Image denoising methods can be divided into two categories: model-based methods and learning-based methods. Many traditional methods belong to the former category (e.g., BM3D [24], where nonlocal means filter [25]), but they suffer from several problems: the need to set parameters manually, and the models can only be targeted at a single denoising task.

Although learning-based methods include some dictionary-based methods, such as [26], the current trend is neural network-based methods. Zhang et al. proposed a feedforward denoising convolutional neural network DnCNN [7], including residual learning (RL) [27] and batch normalization (BN) [28] to restore damaged images, achieving an effective denoising discriminant model. However, deep networks may cause performance degradation as the depth increases. In order to solve this problem, Zhang et al. proposed a fast and flexible denoising model, FFDNet [8], that can handle spatial variation noise quickly and effectively. Tian et al. proposed an attention-guided denoising convolutional neural network, ADNet, for denoising [9]. DudeNet [10] uses a sparse dual denoising network to extract complementary features and fuse global and local features to restore noisy images. MWDCNN [11] uses a combination of signal-processing techniques and discriminative learning to suppress noise and recover more details. However, the above methods focus on continuously improving the network structure and removing Gaussian noise, ignoring the generalization ability of the denoising model.

On the other hand, many methods attempt to model mixed Poisson–Gaussian noise in infrared imagery, training this by adding post-modeled noise [14,15,16]. Kuang et al. used a GaN-based method to denoise infrared images [29]. Xiao et al. proposed a deep residual network architecture to remove noise from meteorological satellite infrared cloud images [30]. Zhang et al. developed an infrared star image-denoising model in which an iterative denoising process is performed on the star area based on deep reinforcement learning [31]. Since the noise in terahertz imagery is difficult to model, the authors of ref. [17] proposed a nonlocal averaging method to denoise the images by adding noise and denoising.

In contrast with previous methods, we propose a pixel-wise crossmodal image-denoising method based on deep reinforcement learning, translating the denoising problem into an iterative and progressive process. We try to construct a simple pixel-wise action sequence to achieve crossmodal image denoising, which can not only remove Gaussian noise in RGB images but also process the complex noise in infrared and terahertz images effectively.

## 3. Methodology

**Figure 2:**
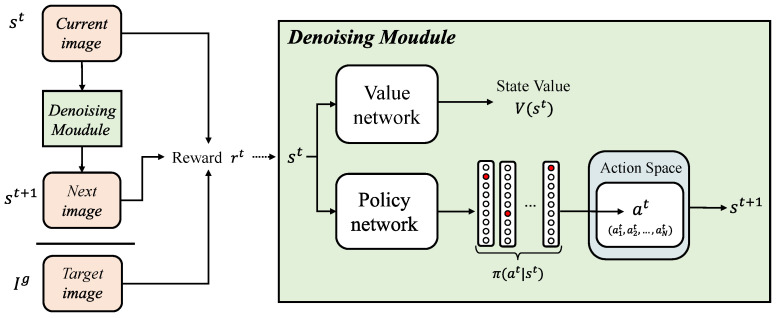
The overall architecture of the proposed method. At the *t*-th step, given the image $s^{t}$, the agents execute the action $a^{t}$ to generate the next image, $s^{t+1}$, according to the strategy $\pi (a^{t}|s^{t};\theta_{p})$ is generated by the policy network; it then uses the current image $s^{t}$, the next image $s^{t+1}$, and the target image $I^{g}$ to calculate the similarity reward, which is used to train the denoising module to help optimize the strategy of the next step.

We propose a pixel-wise crossmodal image-denoising framework based on deep reinforcement learning, which decomposes the denoising problem into an iterative and progressive process. Specifically, we treat the task process as a Markov decision process and decompose it into a series of iterations. Each pixel is treated as an agent, selecting an appropriate action based on the policy generated by the policy network in each iteration and then using the obtained similarity reward to optimize the policy. Finally, an optimal strategy that maximizes the similarity reward of all pixels is learned, achieving progressive denoising. The overall architecture of the proposed method is shown in Figure 2.

### 3.1. Pixel-Wise Reinforcement Learning

Considering the successful performance of the asynchronous actor-critic algorithm A3C [32], we developed a pixel-wise image-denoising framework. We treated each pixel in the input image, *I*, represented as $I_{i}$(i=1,2,…,N), as an individual agent. The policy for each pixel was denoted as $\pi_{i}\left(a_{i}^{t}|s_{i}^{t};\theta_{p}\right)$, where $a_{i}^{t}$ represented the action taken by the *i*-th pixel at time step *t*, and $s_{i}^{t}$ represented its corresponding state. This allowed us to apply the A3C algorithm at the pixel level and effectively denoise the image.

A simple solution to achieve pixel-wise reinforcement learning is training a network that generates policies for each individual pixel. However, this is difficult to calculate due to the high dimensionality of the last connected layer, which would need to be ${\left|A\right|}^{N}$, where |A| represents the dimension of the output policy and *N* is the total number of pixels in the image. Another approach is to divide this problem into *N* independent subnetworks and train *N* subnetworks to maximize the reward of each pixel. However, training *N* networks is also computationally difficult when the number of pixels is large.

Therefore, we propose PixelRL [21] uses FCN to replace *N* subnetworks and extends A3C to the fully convolutional form. By using FCN, the *N* agents can share parameters and can be calculated in parallel on the GPU to ensure the efficiency of training.

### 3.2. Policy Generation

The A3C algorithm is composed of a value network and a policy network. The value function, $V(s^{t};\theta_{v})$, output by the value network estimates the expected total discount reward of the current state image, $s^{t}$, indicating the quality of the state, where $\theta_{v}$ represents the corresponding network parameters. The total discounted reward, $R^{t}$, is represented as

(1) $$\begin{array}{c} R^{t}=\sum_{i=0}^{k-1}\gamma^{i}r^{t+i}+\gamma^{k}V\left(s^{t+k};\theta_{v}\right) \end{array}$$

where $\gamma^{i}$ is the *i*-th power of the discount factor γ, $r^{t}$ is the immediate environmental reward (this will be introduced in Section 3.4). The goal of the reinforcement learning task is to maximize the total discounted reward, $R^{t}$, which takes into account the cumulative effect of actions over multiple steps rather than solely focusing on the immediate environmental reward, $r^{t}$. The reason for this is that the actions performed on the environment have a potential impact on future rewards.

The policy network outputs the strategy $\pi (a^{t}|s^{t};\theta_{p})$ for selecting the action $a^{t}$, given the current state, $s^{t}$. In the context of pixel-wise reinforcement learning, the overall strategy $\pi (a^{t}|s^{t};\theta_{p})$ is composed of the strategies for all pixels. Each pixel, treated as an individual agent, generates its own strategy, $\pi_{i}\left(a_{i}^{t}|s_{i}^{t};\theta_{p}\right)$, using a softmax function, where $a_{i}^{t}$ and $s_{i}^{t}$ represent the action and the state of the *i*-th pixel at the *t*-th step.

Correspondingly, the action $a^{t}$ is composed of the actions taken by all pixels in the image. It can be represented as $a^{t}=\left(a_{1}^{t},a_{2}^{t},\ldots ,a_{N}^{t}\right)$. Each $a_{i}^{t}$ belongs to the action space A.S., which will be discussed further in Section 3.3. In order to assess the appropriateness of selecting action $a^{t}$ in state $s^{t}$, the advantage function is defined as follows:

(2) $$\begin{array}{c} A\left(a^{t},s^{t};\theta_{v},\theta_{p}\right)=R^{t}-V\left(s^{t};\theta_{v}\right) \end{array}$$

The advantage function directly quantifies the difference between the performance of action $a^{t}$ and the average performance of all possible actions. A positive value indicates that action $a^{t}$ outperforms the average and is a favorable choice in the given state, $s^{t}$. Conversely, a negative value suggests that action $a^{t}$ performs worse than the average and should be avoided. By comparing the advantage function to a threshold (e.g., 0), we can evaluate the relative effectiveness of different actions and guide the decision-making process in selecting actions.

### 3.3. Action Space

As mentioned in Section 3.2, we treated each pixel in the noisy image as an agent, and they selected the corresponding action from the predefined action space and executed it in each iteration, ultimately achieving iterative denoising. Considering the characteristics of the image-denoising task, we decided to use predefined discrete actions to form the action space. Our goal is to obtain a model that better handles the results of crossmodal noisy images. To this end, we designed a set of discrete actions to construct the action space, which can handle multiple noise types in different modalities and effectively achieve crossmodal image denoising.

The action space we designed consists mainly of a series of filters, including a Gaussian filter, bilateral filter, guided filter, median filter, and box filter. The Gaussian filter smoothes the image by applying a Gaussian kernel. It is a commonly used linear smoothing filter that can effectively reduce high-frequency noise, especially Gaussian noise. The bilateral filter considers the spatial distance between pixels and the difference between pixel values when performing smoothing processing. The guided filter uses the structural information of the guided image to guide the filtering process, maintains the edge information of the image, and avoids edge blur and detail loss. Both can retain the edge information of the image and have better removal effects on various noise types, such as Poisson noise. The median filter uses a sliding window to find the median value in the neighborhood around the pixel and replaces the original value of the pixel with the median value. It usually has a good denoising effect on peak noise and periodic noise, such as salt and pepper noise. The box filter smoothes the image by calculating the neighborhood average around the pixel, which is a simple and computationally efficient filter.

The parameters for these operations are set based on empirical values. In addition, a “PASS” action has been added. The agents can choose the “PASS” action instead of performing a denoising operation, avoiding excessive denoising to help improve the model’s performance. Table 1 shows the list of operations that an agent can perform.

### 3.4. Reward

For pixel-wise reinforcement learning, the overall reward of the image is composed of the total reward of all pixels in the image. Therefore, our training goal is to learn an optimal policy to maximize the total reward of all pixels, that is, to maximize the mean of the total rewards of all pixels at the *t*-th step. In order to facilitate calculation, we used the mean of the total reward of all pixels at the *t*-th step as the immediate environmental reward obtained by the entire image at the *t*-th step:

(3) $$\begin{array}{c} r^{t}=\frac{1}{N}\sum_{i=1}^{N}r_{i}^{t} \end{array}$$

where *N* represents the number of pixels in the image, and $r_{i}^{t}$ represents the immediate environmental reward obtained by the *i*-th pixel of the noise image $s^{t}$ at the *t*-th step.

The target image is defined as $I^{g}$, and we defined a distance to describe the difference of the *i*-th pixel between the current state $s^{t}$ and the target image:

(4) $$\begin{array}{c} d\left(s_{i}^{t}\right)=\left|\right|I_{i}^{g}-s_{i}^{t}{\left|\right|}_{2} \end{array}$$

where $I_{i}^{g}$ and $s_{i}^{t}$ represent the *i*th pixel value of the original clean image and the current state image, respectively. The distance $d(s_{i}^{t})$ intuitively shows the difference between the *i*-th pixel of the current state image and the *i*-th pixel of the target image, which means how much the error on the *i*-th pixel was decreased by action $a_{i}^{t}$. For a certain pixel, the greater the distance, the further the current image is from the target.

Based on the above analysis, for a specific pixel, we take the difference between the distance $d(s_{i}^{t})$ and the distance $d(s_{i}^{t+1})$ as the environmental reward of the pixel at the *t*-th step. Finally, the similarity reward of the entire image at the *t*-th step is further defined as

(5) $$\begin{array}{c} r^{t}=\frac{1}{N}\sum_{i=1}^{N}\left(d\left(s_{i}^{t}\right)-d\left(s_{i}^{t+1}\right)\right)=\frac{1}{N}\sum_{i=1}^{N}\left(|\right|I_{i}^{g}-s_{i}^{t}{\left|\right|}_{2}-\left|\right|I_{i}^{g}-s_{i}^{t+1}{\left|\right|}_{2}) \end{array}$$

Maximizing the similarity reward $r^{t}$ has the effect of minimizing the distance between the final state and the target image.

## 4. Experiment

### 4.1. Experimental Setting

#### 4.1.1. Training Details

We used the Waterloo exploration database [33] for training and testing, which consists of 4744 original natural images and 94,880 distorted images created from these; we only used the normal images from it. We set the batch size to 16 and performed 70 × 70 random cropping, left-right flipping, and random rotation augmentation on the training images. Our framework was trained using PyTorch on an NVIDIA 2080Ti GPU (NVIDIA, Santa Clara, CA, USA) and used the ADAM optimizer [34] with a starting learning rate of $1.0\times 10^{-3}$ and a minimum learning rate of $1.0\times 10^{-5}$. The maximum number of iterations of training was 30,000, and the number of steps in each episode was set to six.

#### 4.1.2. Metrics

Image quality assessment (IQA) is required to evaluate the quality of the resulting images. The relatively fair and mainstream evaluation indicators in image denoising are the full reference indicators PSNR and SSIM, which require reference images. Considering that some infrared and terahertz images do not have corresponding reference images, in addition, we used the no-reference-image quality evaluation indicators NIQE [35] and PIQUE [36] to evaluate the quality of the resulting images.

**PSNR.** This is the peak signal-to-noise ratio, which is widely used in low-level vision tasks. The value of PSNR is always non-negative, and the larger the value, the better the quality of the image. This value is infinite when the image to be evaluated is the same as the reference image. The specific expression is as follows:

(6) $$PSNR=10lg(\frac{{MAX}^{2}}{MSE})$$

(7) $$MSE=\frac{1}{MN}\sum_{i=0}^{M-1}\sum_{j=0}^{N-1}{\left[I\left(i,j\right)-K\left(i,j\right)\right]}^{2}$$

where MAX represents the maximum pixel value in the image, *M*, *N* denotes the size of the image, and (i,j) denotes the co-ordinates of the pixel points.

**SSIM.** Structural similarity measures image similarity by using image brightness (l), contrast (c), and structure (s). The formula is as follows:

(8) $$SSIM\left(x,y\right)={\left[l\left(x,y\right)\right]}^{\alpha }{\left[c\left(x,y\right)\right]}^{\beta }{\left[s\left(x,y\right)\right]}^{\gamma }$$

where α, β, and γ are parameters that are frequently set as being equal to 1, and *x* and *y* represent the two input images. The range of SSIM is [0,1]. When the image to be evaluated is completely identical to the reference image, the value is 1. SSIM has significantly higher computational complexity than PSNR, but it can contain more image-quality information.

**NIQE.** Natural image quality evaluator is a model that is designed to assess the quality of images by constructing a set of features that measure image quality and fit them into the multivariate Gaussian model. The NIQE value quantifies the discrepancy between an image and a standard natural image, where a higher NIQE value indicates poorer image quality.

**PIQUE.** Perception-based image quality evaluator is a no-reference image quality assessment metric that does not require training. A lower score indicates better perceptual quality.

### 4.2. Experiments on RGB Images

In this section, we demonstrate the denoising effect of RGB images after adding AWGN. In order to compare the performance of our proposed method, we compared it with several SOTA methods: DnCNN [7], FFDNet [8], ADNet [9], DudeNet [10], and MWDCNN [11]. We retrained the methods mentioned above according to the setting and conducted a series of comprehensive experiments, including visual quality comparison and image quality evaluation, to qualitatively and quantitatively analyze the denoising performance of the proposed model.

#### 4.2.1. Visual Quality Comparison

**Figure 3:**
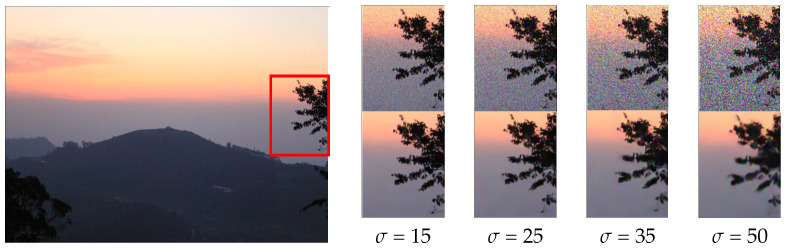
An example image of different noise levels (15, 25, 35, and 50) and the denoising results of our method under the corresponding noise levels. The top line represents the area of the noisy image, and the bottom line represents the result after denoising.

**Figure 4:**
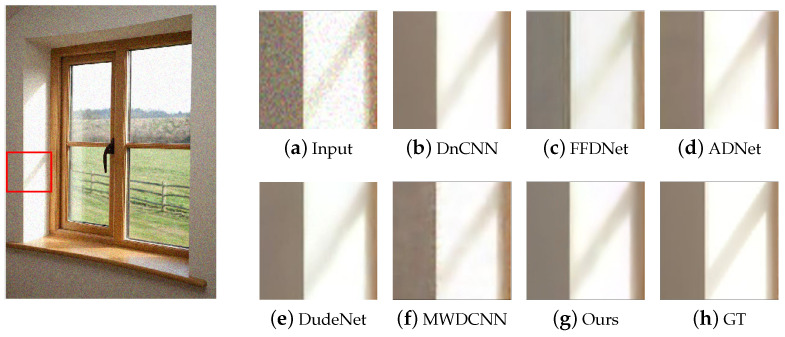
The results of different methods on the test dataset with σ = 15. GT stands for ground truth, which is the clean image.

In terms of qualitative analysis, we used the images in the Waterloo exploration database [33] as test data for denoising to compare visual denoising performance. For the predicted images of different methods, an area can be selected and enlarged as an observation area. The observation area is clearer, and the corresponding denoising method is more effective for image denoising. We test the denoising performance with the noise levels of 15, 25, 35, and 50, and Figure 3 shows an example image with different noise levels and the denoising results of our methods. Figure 4 shows the visual experimental results compared with other methods.

It can be seen that DnCNN does lose some details; FFDNet needs to improve the effect on details in the light and shaded areas; ADNet and DudeNet have a certain degree of blur and distortion; MWDCNN produces blocking artifacts, although some details are preserved. In contrast, our method preserves details while minimizing blurring and distortion as much as possible, which achieves the best visual effect.

#### 4.2.2. Image Quality Assessment

In order to conduct a comprehensive quantitative comparison, we used PSNR and SSIM to evaluate the quality of the denoising results objectively. We tested the denoising performance of different methods with noise levels of 15, 25, 35, and 50, and the results are shown in Table 2.

Although our method is only optimal in PSNR when the noise level is 15, it has a good overall effect on Gaussian noise denoising in RGB images, and we achieve a balance between the PSNR and SSIM values.

### 4.3. Experiments on Infrared Images

We conducted qualitative and quantitative comparisons on two public infrared datasets: the paired infrared denoising database (http://openai.raytrontek.com/, accessed on 8 April 2022) and the pedestrian dataset (the OSU thermal pedestrian database) [37]. The former is a real-world infrared image-denoising database proposed by the Robotics and Artificial Intelligence Laboratory of IRay Technology Co., Ltd. (Yantai, China). It contains 2000 low-quality and high-quality infrared image pairs in different scenarios, and the content is mainly indoor scenes. Since there are many images with repeated scenes in the paired infrared denoising dataset, we selected 100 typical images to form a test dataset called “Pair100”. The OSU thermal pedestrian database contains 284 noisy infrared images in 10 subsets, with common outdoor scenes, pedestrians, and vehicles as the main content. We compared this with DnCNNb [7] (DnCNNb is the model trained by blind denoising), FFDNet [8], ADNet [9], DudeNet [10], MWDCNN [11], HI-GAN [38], and LG-BPN [39]. Moreover, we additionally conducted experiments using DnCNN15, DnCNN25, and DnCNN50 (DnCNN15 is the model trained with a noise level of 15, and the same applies later).

#### 4.3.1. Visual Quality Comparison

**Figure 5:**
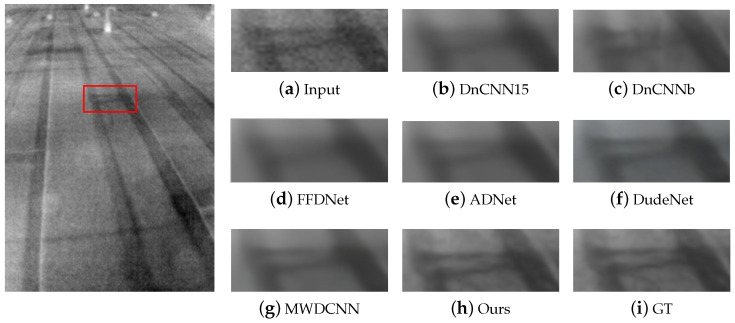
The results of different methods on image #0941 in Pair100. GT stands for ground truth, which is the clean image. The red box indicates the observation area that needs to be enlarged.

**Figure 6:**
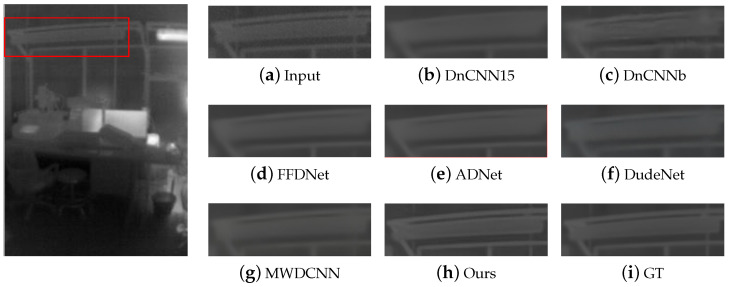
The results of different methods on image #0546 in Pair100. GT stands for ground truth, which is the clean image. The red box indicates the observation area that needs to be enlarged.

**Figure 7:**
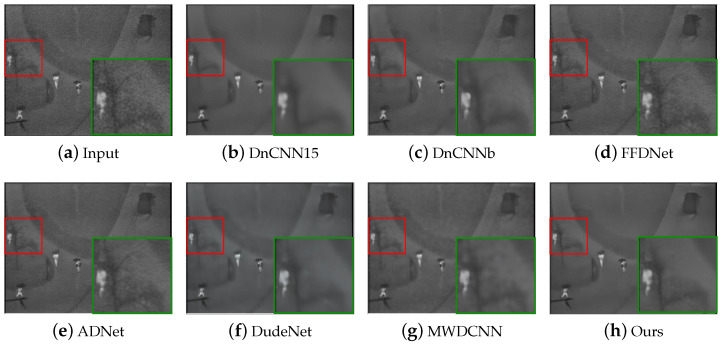
The results of different methods on image #00001 in the subset 00001 of the OSU thermal pedestrian database. The red box indicates the observation area that needs to be enlarged, and the green box indicates the enlarged result of the corresponding area.

**Figure 8:**
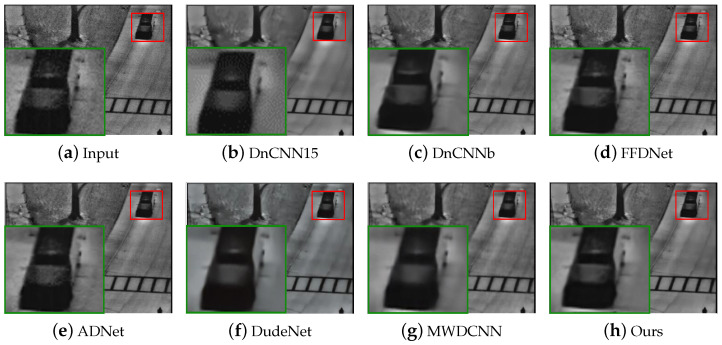
The results of different methods on image #00004 in the subset 00003 of the OSU thermal pedestrian database. The red box indicates the observation area that needs to be enlarged, and the green box indicates the enlarged result of the corresponding area.

In terms of qualitative analysis, we conducted denoising tests using two infrared datasets to compare visual denoising performance. Figure 5 and Figure 6 show the visualization results in Pair100, and the visualization results in the OSU thermal pedestrian database are shown in Figure 7 and Figure 8.

It can be observed that DnCNN is prone to excessive denoising, resulting in the loss of some details, and DnCNN15 produces more blur artifacts compared to DnCNNb; FFDNet and ADNet handle some noise poorly and retain more noise; DudeNet has more blur, while MWDCNN produces some distortions and artifacts. Compared with the above methods, our method avoids excessive blurring and artifacts and retains details as much as possible while removing noise.

#### 4.3.2. Image Quality Assessment

For the quantitative experiments, since Pair100 provides corresponding high-quality images, we used PSNR and SSIM as evaluation metrics. The experimental results are shown in Table 3. It can be seen that our method achieves the best PSNR and SSIM values compared to other methods trained on RGB images using AWGN.

However, the OSU thermal pedestrian database does not have corresponding high-quality images and cannot be evaluated using the PSNR and SSIM indicators. In order to evaluate the quality of the results directly, we used NIQE and PIQUE. The evaluation was performed separately on 10 subsets of the OSU thermal pedestrian database, and the results are shown in Table 4 and Table 5. It can be seen that our method obtains optimal or suboptimal results in most subsets, and on average, our NIQE is the best, and PIQUE is suboptimal.

### 4.4. Experiments on Terahertz Images

This section mainly presents our experimental results on terahertz images. We selected the terahertz images provided in the terahertz dataset [40] as the test dataset. The dataset contains two parts used for target detection and segmentation, respectively. We randomly selected 800 images from the first part containing 3165 images to construct the test dataset.

#### 4.4.1. Visual Quality Comparison

**Figure 9:**
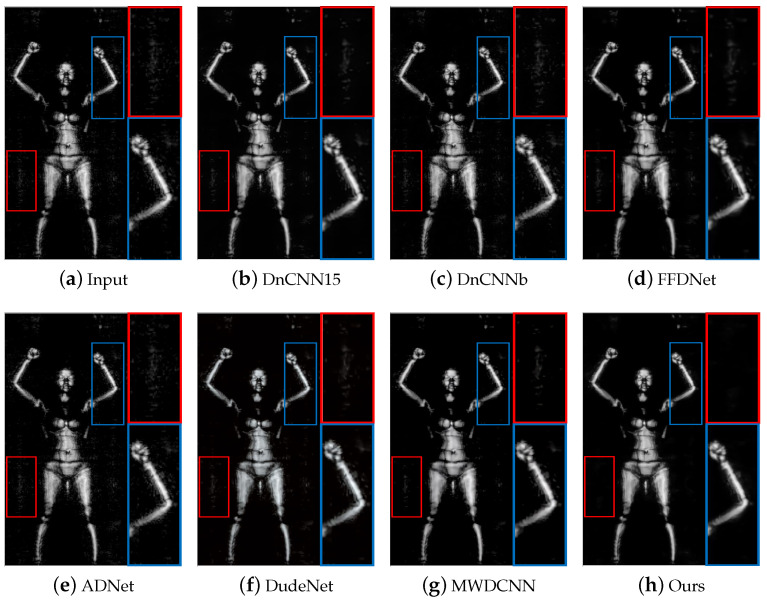
The results of different methods on image #D_N_F1_CK_F_LA_WB_F_S_front_0907140917 in the terahertz test dataset. The red/blue box represents the observation area that needs to be enlarged, and the same color box on the right represents the result of enlarging the corresponding area.

**Figure 10:**
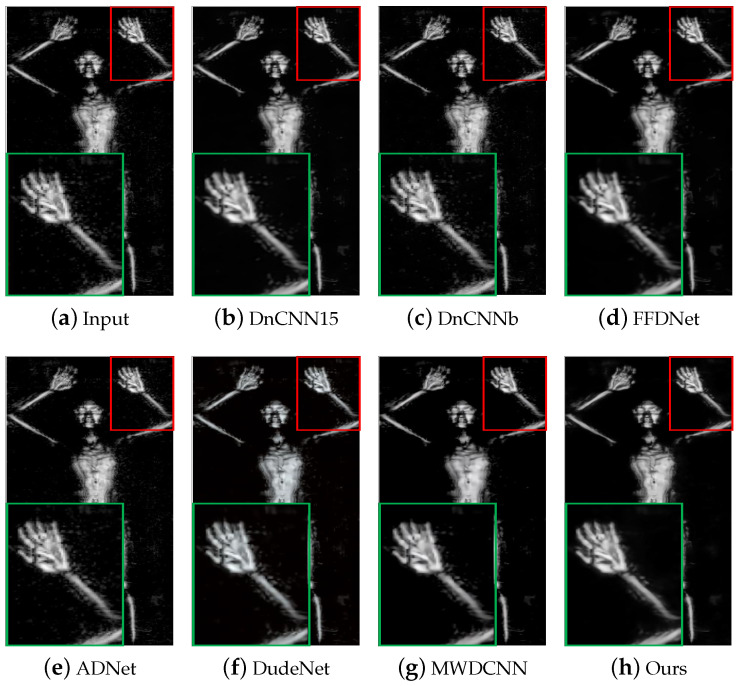
The results of different methods on image #D_N_M1_GA_F_RT_KK_F_W_front_0903164009 in the terahertz test dataset. The red box indicates the observation area that needs to be enlarged, and the green box indicates the enlarged result of the corresponding area.

Figure 9 and Figure 10 show the visual experimental results of some experiments. For the obvious noise blocks in the terahertz image (e.g., the part inside the red box in Figure 9), the comparison methods have a poor removal effect and retain some noise, but our method can effectively remove them. Moreover, for spot-like noise around the human body (e.g., the part inside the box in Figure 10), our method also achieves better removal results than other methods.

#### 4.4.2. Image Quality Assessment

Since the terahertz images do not have corresponding high-quality images, we used NIQE and PIQUE to evaluate the quality of the results directly. The evaluation results are shown in Table 6 and Table 7. The results show that our method achieves relatively good results overall compared to other methods.

### 4.5. Ablation Study

Action space is important in deep reinforcement learning as it defines all possible actions that an agent can take, and the design of the action space has a direct impact on the performance and effect of the reinforcement learning algorithm. Considering the characteristics of the denoising problem, we constructed the action space as a series of filters as discrete actions. However, using only filters as actions will likely result in excessive denoising. To this end, we added a “PASS” action, allowing the agents to choose not to perform denoising operations to prevent excessive denoising. In order to verify the effectiveness of the “PASS” action we added, we conducted an ablation experiment. Table 8 shows the corresponding results.

**Figure 11:**
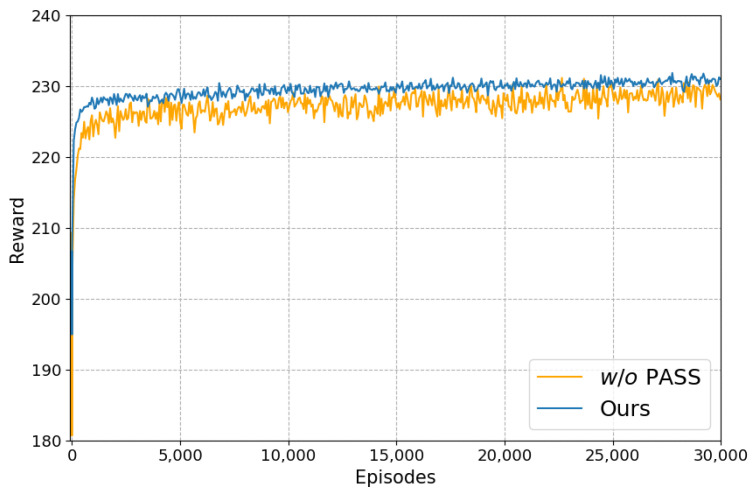
The cumulative reward value obtained by all pixels after six steps of each episode during training (σ = 25).

It can be seen that after adding the “PASS” action, the PSNR and SSIM under four noise levels have been significantly improved. Figure 11 shows the training curves of the method during ablation experiments, demonstrating the convergence of the algorithm training process. From Figure 11, we can see that the cumulative reward value obtained by all pixels after six steps of each episode rises dramatically and stabilizes eventually with the increase in episodes. Moreover, the reward will remain at a relatively higher value and be more stable after adding the “PASS” action, which also proves the necessity of the “PASS” action.

### 4.6. Discussion

We propose a pixel-by-pixel crossmodal image-denoising method that leverages a reinforcement learning framework to view the image-denoising task as a series of iterative decision-making processes, further treating it as a Markov decision process. By treating each pixel as an agent, the policy network and the value network are used to learn the optimal policy to maximize the similarity reward between pixels and, ultimately, obtain the optimal result. In addition, considering the lack of high-quality datasets for infrared and terahertz images and the difficulty in modeling noise, which leads to difficulty in training, we trained a crossmodal model to achieve the effective denoising of infrared and terahertz images. The experimental results also demonstrate the effectiveness of our method.

However, our method still has certain limitations. After considering the simplicity and effectiveness of the basic filters, we chose to design an action set based on the basic filters as the action space. However, the basic filters are limited in their effectiveness in dealing with noise, and using only the filters mentioned in the text may limit the effectiveness of our method in dealing with certain types of noise or scenes, meaning that the settings for the action space need to be further improved.

## 5. Conclusions

This paper proposes a pixel-wise crossmodal image-denoising method based on reinforcement learning, which can effectively denoise images in different modalities. We regard the task process as a Markov decision process and treat each image pixel as an agent, learning an optimal strategy to maximize the similarity reward of all pixels to obtain the optimal result. In addition, we designed a set of actions with which to build the action space and define the similarity reward to train the policy network and value network to help obtain optimal results. We demonstrate the effectiveness of our method in RGB, infrared, and terahertz image denoising in detail via both visual effects and image-quality evaluations. The experimental results prove that our method can effectively handle noise in three types of images, enhance the generalization performance of the model, and achieve crossmodal image denoising. However, the setting of our action set still needs improvement. Even if the “PASS” action is added, there are still certain limitations in using only the listed filters as denoising actions. Our future work will explore how to set actions in the action space more reasonably and further improve the ability to handle various noises.

## Figures and Tables

**Table 1:** Actions for crossmodal image denoising. We defined nine actions, one of which is “PASS”, and the rest are denoising operations.

	Action	Parameter
1	Gaussian filter	σ = 0.5
2	Gaussian filter	σ = 1.0
3	Bilateral fliter	$\sigma_{c}$ = 0.5, $\sigma_{s}$ = 5.0
4	Bilateral fliter	$\sigma_{c}$ = 1.0, $\sigma_{s}$ = 5.0
5	Guided filter	r = 2, eps = 0.01
6	Guided filter	r = 2, eps = 1.00
7	Median filter	-
8	Box fliter	-
9	PASS	-

**Table 2:** The image quality assessment results (PSNR ↑ and SSIM ↑) on the test dataset, with a noise level of 15, 25, 35, and 50. The bold and underlined text represents optimal and suboptimal results, respectively.

Methods	σ = 15		σ = 25		σ = 35		σ = 50	
	PSNR	SSIM	PSNR	SSIM	PSNR	SSIM	PSNR	SSIM
Noisy	25.085	0.455	20.850	0.289	18.130	0.205	15.349	0.137
DnCNN	34.103	0.907	32.405	0.891	30.703	0.836	28.746	0.782
FFDNet	34.034	**0.927**	32.426	**0.905**	30.756	**0.875**	29.035	**0.841**
ADNet	34.359	0.906	32.613	0.865	31.048	0.837	29.220	0.822
DudeNet	34.130	0.923	32.575	0.877	31.021	0.836	29.265	0.810
MWDCNN	34.600	0.914	**32.816**	0.887	**31.117**	0.856	**29.463**	0.814
Ours	**34.610**	0.923	32.444	0.891	31.052	0.858	29.269	0.827

**Table 3:** The image quality assessment results (PSNR ↑ and SSIM ↑) using Pair100. Dn.15, Dn.25, Dn.50, Dn.b, and D.Net represent DnCNN15, DnCNN25, DnCNN50, DnCNNb, and DudeNet, respectively. The bold and underlined text represents the optimal and suboptimal results, respectively.

	Dn.15	Dn.25	Dn.50	Dn.b	FFDNet	ADNet	D.Net	MWDCNN	HI-GAN	LG-BPN	Ours
PSNR	33.595	32.742	30.587	34.453	34.599	34.736	34.878	35.102	36.094	36.226	**36.473**
SSIM	0.929	0.909	0.878	0.939	0.939	0.936	0.931	0.938	0.940	0.948	**0.951**

**Table 4:** The image quality assessment results (NIQE ↓) using 10 subsets of the OSU thermal pedestrian database. Dn.15, Dn.25, Dn.50, Dn.b, and D.Net represent DnCNN15, DnCNN25, DnCNN50, DnCNNb, and DudeNet, respectively. The bold and underlined text represents the optimal and suboptimal results, respectively.

	Dn.15	Dn.25	Dn.50	Dn.b	FFDNet	ADNet	D.Net	MWDCNN	HI-GAN	LG-BPN	Ours
00001	14.497	16.380	17.030	8.023	7.847	7.903	10.994	8.187	8.161	7.789	**7.758**
00002	14.766	16.167	17.436	7.651	**7.013**	7.697	12.136	7.841	7.544	7.967	7.588
00003	6.319	8.252	8.538	4.672	4.697	**4.511**	4.736	5.335	5.217	6.602	4.925
00004	11.816	15.069	15.302	6.694	6.577	6.659	9.885	7.654	**5.654**	7.579	6.466
00005	9.159	10.974	12.644	**4.581**	5.049	5.241	6.006	6.577	6.511	6.806	4.945
00006	14.147	14.164	14.860	6.927	6.869	6.959	10.789	6.840	6.837	7.175	**6.805**
00007	15.373	16.588	19.235	9.345	9.059	8.391	13.215	**8.308**	10.765	8.332	9.284
00008	15.236	16.519	16.795	8.002	7.931	7.958	10.955	7.723	7.255	**6.763**	7.903
00009	13.527	14.405	16.285	7.626	**6.387**	7.597	9.974	7.385	7.682	7.733	7.609
00010	11.013	12.495	14.731	4.933	6.455	5.862	7.994	6.516	**4.920**	6.073	5.743
Avg	12.553	14.112	15.857	6.812	6.795	6.884	9.682	7.268	6.803	7.115	**6.767**

**Table 5:** The image quality assessment results (PIQUE ↓) using 10 subsets of the OSU thermal pedestrian database. Dn.15, Dn.25, Dn.50, Dn.b, and D.Net represent DnCNN15, DnCNN25, DnCNN50, DnCNNb, and DudeNet, respectively. The bold and underlined text represents the optimal and suboptimal results, respectively.

	Dn.15	Dn.25	Dn.50	Dn.b	FFDNet	ADNet	D.Net	MWDCNN	HI-GAN	LG-BPN	Ours
00001	86.209	89.675	89.287	47.320	34.046	33.747	33.357	32.075	59.734	76.591	**31.988**
00002	84.583	88.743	88.697	47.663	**34.538**	44.918	42.089	41.697	52.183	69.133	39.980
00003	81.940	86.585	86.815	54.777	**40.347**	46.353	66.457	66.380	50.314	68.435	52.591
00004	85.556	88.716	88.284	48.328	**35.137**	46.357	46.342	41.192	51.144	69.963	44.076
00005	85.701	88.255	88.955	49.625	**36.491**	38.322	52.226	48.792	46.290	68.823	44.998
00006	86.527	87.968	89.038	51.646	40.571	35.967	**30.122**	38.946	51.667	57.409	32.029
00007	88.105	91.253	89.869	49.482	36.585	39.534	**31.672**	33.221	69.659	76.610	41.177
00008	89.383	91.191	89.795	51.749	45.038	**36.700**	47.205	45.234	58.611	69.883	44.596
00009	90.022	90.275	89.336	42.780	**31.067**	34.068	34.536	34.825	52.194	64.987	38.942
00010	87.471	90.106	89.376	**32.386**	45.213	46.778	50.170	47.730	48.286	64.660	45.073
Avg	85.532	89.632	88.526	47.537	**38.545**	41.562	43.668	42.559	52.479	67.555	40.856

**Table 6:** The image quality assessment results (NIQE ↓) on the Pair100 and terahertz test datasets. Dn.15, Dn.25, Dn.50, Dn.b, and D.Net represent DnCNN15, DnCNN25, DnCNN50, DnCNNb, and DudeNet, respectively. The bold and underlined text represents the optimal and suboptimal results, respectively.

	Dn.15	Dn.25	Dn.50	Dn.b	FFDNet	ADNet	D.Net	MWDCNN	HI-GAN	LG-BPN	Ours
Pair100	14.185	15.670	15.565	8.697	14.731	14.635	11.998	13.664	8.598	8.250	**8.183**
Terahertz	10.196	10.575	10.887	9.836	10.915	10.716	10.928	12.524	9.835	10.221	**9.528**

**Table 7:** The image quality assessment results (PIQUE ↓) on Pair100 and terahertz test datasets. Dn.15, Dn.25, Dn.50, Dn.b, and D.Net represent DnCNN15, DnCNN25, DnCNN50, DnCNNb, and DudeNet, respectively. The bold and underlined text represents the optimal and suboptimal results, respectively.

	Dn.15	Dn.25	Dn.50	Dn.b	FFDNet	ADNet	D.Net	MWDCNN	HI-GAN	LG-BPN	Ours
Pair100	91.054	95.863	97.367	52.171	90.726	43.436	51.493	83.492	65.000	66.073	**40.319**
Terahertz	79.538	82.431	84.674	**76.517**	85.369	79.233	83.024	84.706	80.291	86.135	78.235

**Table 8:** Ablation experiment results on the “PASS” action. The table shows the PSNR ↑ and SSIM ↑ on RGB images with noise levels of 15, 25, 35, and 50. The bold indicates the optimal results.

	σ=15		σ=25		σ=35		σ=50	
	PSNR	SSIM	PSNR	SSIM	PSNR	SSIM	PSNR	SSIM
w/o PASS	34.588	0.920	32.196	0.889	30.524	0.856	28.792	0.817
Ours	**34.610**	**0.923**	**32.444**	**0.891**	**31.052**	**0.858**	**29.269**	**0.827**

## Data Availability

No new data were created or analyzed in this study. Data sharing is not applicable to this article.
